# Using prosocial behavior to safeguard mental health and foster emotional well-being during the COVID-19 pandemic: A registered report protocol for a randomized trial

**DOI:** 10.1371/journal.pone.0245865

**Published:** 2021-01-27

**Authors:** Andrew Miles, Meena Andiappan, Laura Upenieks, Christos Orfanidis

**Affiliations:** 1 Department of Sociology, University of Toronto, Toronto, Canada; 2 Institute of Health Policy, Management, and Evaluation, University of Toronto, Toronto, Canada; 3 Department of Sociology, Baylor University, Waco TX, United States of America; University of Helsinki, FINLAND

## Abstract

The COVID-19 pandemic, the accompanying lockdown measures, and their possible long-term effects have made mental health a pressing public health concern. Acts that focus on benefiting others—known as prosocial behaviors—offer one promising intervention that is both flexible and low cost. However, neither the range of emotional states prosocial acts impact nor the size of those effects is currently clear, both of which directly influence its attractiveness as a treatment option. Using a large online sample from Canada and the United States, we will examine the effect of a three-week prosocial intervention on two indicators of emotional well-being (happiness and the belief that one’s life is valuable) and mental health (anxiety and depression). Respondents will be randomly assigned to perform prosocial, self-focused, or neutral behaviors each week. Two weeks after the intervention, a final survey will assess whether the intervention has a lasting effect on mental health and emotional well-being. Our results will illuminate whether prosocial interventions are a viable approach to addressing mental health needs during the current COVID-19 pandemic, as well for those who face emotional challenges during normal times.

## Introduction

COVID-19 has elicited an unprecedented global response to safeguard the physical health of vulnerable populations around the world. National and local governments have implemented strict measures to minimize contact between individuals in an effort to curb the spread of infection. People have been asked to limit their physical interactions with others, stay inside their homes, and reduce both professional and personal ties that cannot be maintained at a distance. The success of this approach has been encouraging, but the price of isolation on mental health is likely to be high [1–4]. Early evidence from China suggested that these fears were being realized. In the immediate aftermath of lockdown measures, Chinese respondents reported moderate to high levels of stress, as well as high rates of mental health challenges including anxiety, depressive symptoms, and sleep disruption [5, 6]. More recent research conducted in Canadian [7], American [8, 9] and Dutch contexts [10] also show trends toward worsening mental health and higher distress across people of diverse ages as the pandemic unfolds.

There has long been a need to safeguard the mental health and emotional well-being of individuals in precarious circumstances, but the COVID-19 pandemic and its likely social and economic aftereffects have made this need even more pressing. Trained mental health providers are in short supply and likely to remain so as societies begin to experience the long-term effects of the COVID-19 response. There is accordingly a strong need for efficient, effective, and low-cost strategies for preserving mental health that can be deployed rapidly and widely. One highly portable solution would be to develop self-guided and home-based interventions [11, 12].

Acts that focus on benefiting others—known as prosocial behaviors—offer one promising approach. The benefits of prosocial activities have been well-supported in the literature. Prosocial acts have been shown to boost a number of mental states including life satisfaction, well-being, and psychological flourishing. These effects can last for several weeks or even months following the end of an intervention [13–18]. Evidence indicates that prosocial behaviors produce positive emotions and happiness even when performed at a distance, making them ideally suited to the current crisis [19–21]. Prosocial acts can be flexibly enacted in many circumstances and often at little to no cost, facts which give a prosocial intervention the potential to be implemented quickly and widely.

There are two major limitations to past work that must be addressed before prosocial activity can be recommended to address the mental health concerns associated with COVID-19. First, past research has focused overwhelmingly on positive affect and happiness. Less attention has been given to other positive states such as a sense of meaning in life, and to negative states including anxiety and depression, though initial work along these lines is promising. More fully establishing prosocial behavior’s impact on outcomes such as these is necessary to fully understand its therapeutic potential. Second, current knowledge about the effectiveness of prosocial action is based predominately on small-sample studies that are likely underpowered to detect effects. Post-hoc efforts to correct for this fact suggest that the true effects of prosocial acts on emotions might be smaller than previously supposed [22, 23]. It thus remains unclear 1) how large and 2) how extensive the effects of prosocial behavior are, both of which have direct implications for its attractiveness as a treatment option. This study will address both of these challenges by examining the effect of prosocial behavior on two indicators of emotional well-being (happiness and belief that one’s life if valuable) and mental health (anxiety and depression) during a three-week intervention in a large online sample with sufficient power to detect effects. Because lasting effects have considerable practical appeal, we focus in particular on whether effects persist both throughout the intervention period and at a five-week follow-up.

### Prosocial behavior’s effects on emotional well-being and mental health

Doing good feels good. In the last several decades, this simple maxim has been placed under scientific scrutiny and accumulated a sizable body of evidence that attests to its veracity. Two recent meta-analyses found that prosocial activities produce a small positive effect on “emotional well-being”—a catch-all term that includes happiness, eudaimonic well-being, positive affect, psychological flourishing, and the absence of negative emotions [20, 24]. Prosocial effects have been observed among children as well as adults and in samples across the world [20, 25–30]. Aknin et al. [25] speculate the “warm glow” of giving might be a universal component of human psychology.

The emotional benefits of giving suggest that prosocial acts can be used in interventions to improve mental health. However, the practical utility of prosocial behavior depends on its effectiveness relative to other possible activities. We propose that people who are suffering from low levels of positive emotion and/or high levels of negative emotion often default to doing nothing—that is, they continue to perform routine daily activities that are affectively neutral but neglect to engage in activities intended to improve their emotional well-being. Alternately, they try to improve their mood by engaging in personally enjoyable activities intended to gratify their own emotional needs. We accordingly suggest that the practical utility of prosocial behavior is directly tied to its ability to enhance emotional well-being and relieve mental distress relative to these comparison points.

The majority of research on prosocial behavior and emotions has examined happiness or indices of positive affect (e.g., the Positive and Negative Affect Schedule). Fewer studies have examined the effects of kind acts on other forms of emotional well-being or specific mental health indicators. Our study replicates past work by examining prosocial effects on happiness, and then extends it by contributing to the small body of research assessing prosocial effects on three additional outcomes: sense of meaning in life, anxiety, and depression.

#### Happiness

Work to date indicates that prosocial behaviors produce greater emotional well-being relative to a neutral or no action control condition [16, 31, 32]. This may be because prosocial acts fulfill basic psychological needs for autonomy, competence, and relatedness [33, 34], or possibly a need for morality [35]. Kind acts might also prompt positive thoughts and additional positive behaviors that further enhance well-being [14, 36]. Although the mechanisms underlying prosocial effects on happiness are still being established, the basic relationship appears robust. We accordingly hypothesize that *prosocial acts will increase happiness relative to affectively neutral acts* (Hypothesis 1a).

Prosocial behavior may also have advantages over self-focused acts of personal gratification. Common wisdom suggests that self-gratification should generate positive emotions, and existing research bears this out [37–39]. However, studies of prosocial acts indicate that prosocial behaviors produce greater gains in positive emotion than self-focused actions. For example, those who give to others instead of themselves report higher rates of happiness, regardless of the amount of money or size of the gifts involved, and regardless of the source of the funds [40–43]. Similarly, those who perform kind acts for others enjoy greater emotional well-being than those engaging in self-focused acts [16]. The greater emotional benefits of prosocial purchases, in particular, might reflect the fact that money spent on experiences tend to produce larger and longer-lasting gains in happiness than material purchases [44, 45]. Prosocial spending effects could accordingly arise because they produce positive experiences rather than the acquisition of material goods. Another possibility is that individuals performing personally enjoyable acts might see themselves as self-indulgent, leading to mixed emotions—enjoyment from the self-gratification, but also negative emotions like guilt from a perceived sense of selfishness [46, 47]. Regardless of the underlying mechanisms, we suggest that *prosocial acts will increase happiness relative to self-focused acts intended to gratify personal emotional needs* (Hypothesis 1b).

#### Sense of meaning

Humans have a desire to perceive and preserve meaning in life [48, 49]. Meaning in life can be divided into (at least) three aspects: coherence (life makes sense), purpose (direction and goals in life), and significance (life is valuable and worth living) [50, 51]. While there are reasons to suppose that prosocial action might influence any of these aspects, we focus on significance because we suspect that evaluations of one’s life are more responsive to changing life circumstances—such as those brought on by the COVID-19 pandemic—than are the beliefs that allow individuals to make sense of life, or the long-term goals they hold. Additionally, far less research has examined significance, making the need to understand it more acute [50].

According to Baumeister [52], people find a sense of meaning when they believe that their actions are “right and good and justifiable” (p. 36). Meaning might also arise from the sense of belonging that accompanies positive social connections [50, 53, 54]. Prosocial acts could generate a sense of meaning through either mechanism: they are widely considered to be “right and good”, and they could initiate positive interactions that lead to lasting social connections.

Empirical evidence linking prosocial acts to meaning in life is limited, but consistent with these claims. For example, recent studies by Van Tongeren, Green, Davis, Hook and Hulsey [55] found that engaging in altruistically motivated prosocial behavior is associated with greater meaning in life. These studies used undergraduate student samples, but the same effects have been found in the few studies that have examined adult samples [56, 57]. Although a close examination of these studies reveals that they predominately examined purpose in life rather than significance, the positive correlation between these two aspects suggests that significance will respond to prosocial actions in similar ways [58]. We therefore hypothesize that *prosocial acts will increase a sense that one’s life has value relative to affectively neutral acts* (Hypothesis 2a).

Self-focused acts are unlikely to offer the same benefit. Although these behaviors can produce positive emotions, we suspect that they will not typically be viewed as “right and good.” More often, they will be perceived as morally neutral or even morally suspect to the extent that they are seen as selfish. Self-focused behaviors are also unlikely to lead to interpersonal connections that foster a sense of meaning in life, nor to a sense of attachment to something greater than oneself, which would seem to require attending to concerns beyond the self. We therefore hypothesize that *prosocial acts will increase a sense that one’s life has value relative to self-focused acts intended to gratify personal emotional needs* (Hypothesis 2b).

#### Depression

There are several reasons to expect that prosocial behavior will reduce depressive symptoms. Raposa and colleagues [59] argue that engaging in prosocial behavior can offset the impact of daily life stress on negative affect, which is a hallmark of depression. Depressed individuals also frequently hold negative views of themselves, such as the belief that they are unworthy or ineffective [60]. Prosocial actions could alleviate such judgments by shifting attention away from the self and towards the needs of others. Acts of personal gratification are unlikely to have this effect because they direct focus toward the self. Moreover, research suggests that symptoms of depression include increased interpersonal sensitivity and fear of social disapproval, both of which might prompt difficulty in interacting with others [61, 62]. Engaging in acts of kindness towards others may lessen such concerns. Indeed, some evidence suggests that prosocial behaviors promote social integration and bonding with others [63], which may in turn prompt reciprocal supportive acts that could alleviate fears and reduce depression [64]. Self-focused acts might not confer this benefit because they often occur in isolation and are unlikely to prompt positive reciprocating behavior from others.

A handful of studies provide initial evidence that prosocial acts reduce depressive symptoms. Two studies indicate that individual who regularly engage in kind acts feel better on days when they help strangers [59] or friends [65]. A large cross-national study further found that those who volunteered reported lower levels of depression than those who did not [66]. In two experimental studies, engaging in kind or compassionate acts decreased depression relative to those in an affectively neutral control condition. These benefits persisted from one month to six months following the end of the intervention [67, 68]. Given existing evidence and the theoretical considerations offered above, we hypothesize that *prosocial acts will reduce depression relative to both affectively neutral acts* (Hypothesis 3a) *and self-focused acts intended to gratify personal emotional needs* (Hypothesis 3b).

#### Anxiety

Prosocial acts could also reduce anxiety. Taylor, Lyubomirsky, and Stein [69] noted that positive emotions can reduce the physiological and psychological impact of negative emotions and argued that positive emotions might therefore be an effective treatment for depression and anxiety. Using a sample of individuals suffering from depression or anxiety, they found that those who engaged in positive activities—including prosocial acts—experienced significant improvements in levels of anxiety and depression compared to a wait-list control group. Similar results have been found in other samples of anxious individuals. In these studies, individuals who performed kind acts saw improvements in anxiety and a reduction in social avoidance, an anxiety-related behavior [13, 70, 71]. In contrast, self-focused behavior has been associated with increases in anxiety over time [72]. This may be because self-focused acts can elicit negative emotional reactions such as anxiety, sadness, or guilt if individuals believe that they should not be focusing on themselves or using valuable resources to satisfy their own desires [16, 47]. Existing evidence therefore suggests that prosocial acts can reduce anxiety and might be more effective at doing so than self-focused acts. We therefore hypothesize that *prosocial acts will reduce anxiety relative to both affectively neutral acts* (Hypothesis 4a) *self-focused acts intended to gratify personal emotional needs* (Hypothesis 4b).

The foregoing discussion suggests that there are both theoretical and empirical reasons to believe that prosocial behavior will enhance emotional well-being and improve mental health. However, in several key regards the evidence for prosocial effects is still quite thin. In some cases, this is an issue of quantity: there are relatively few studies linking prosocial behavior to sense of meaning, depression, and anxiety, for instance, so it is difficult to know how robust these results are. Further, a number of these studies use undergraduate students or samples selected for depression or anxiety, so it is unclear how well results generalize to other populations. The same cannot be said of work on prosocial behavior and happiness, which has been examined repeatedly in variety of samples. A second issue, however, applies equally to studies of all outcomes, including happiness. Studies of prosocial effects frequently rely on relatively small sample sizes (e.g., N per condition < 100), and so are likely underpowered. Underpowered studies increase the chance of observing inflated effect sizes because the increased uncertainty in estimates means that only large effects will reach statistical significance [73]. Thus, while the available evidence suggests that prosocial behavior positively influences emotional well-being and improves mental health, it is likely that reported effect sizes are too large. In fact, a recent high-powered replication of prosocial spending research found that effects across three studies ranged from non-existent to modest [24, 43]. Similarly, a recent meta-analysis of positive psychology interventions found that effect sizes were substantially overestimated due to an overreliance on small-N studies [22]. Inflated effect sizes, in turn, have direct implications for the utility of kindness interventions for improving mental health. Our study will address this evidence gap by estimating the effects of kind acts on happiness, sense of meaning, depression, and anxiety in a sample that is large enough to detect even modest effects.

## Materials and methods

Table 1 summarizes key design elements of the study. Refer to the relevant sections of the manuscript for additional details and justifications of study procedures.

**Table 1:** Design table.

Question	Hypothesis	Sampling plan	Analysis Plan	Interpretation given to different outcomes	
Does prosocial behavior increase happiness?	1a. Prosocial acts will increase happiness relative to affectively neutral acts.	**Sample**: Canadian and American respondents from Amazon’s Mechanical Turk.	**Analysis**: four random intercept models of the form:	Hypothesis 1a, 2a, 3a, and 4a confirmed if:	
	1b. Prosocial acts will increase happiness relative to self-focused acts intended to gratify personal emotional needs.	**Design:** between subjects; respondents randomly assigned to complete prosocial acts, self-focused acts, or track activities 3 days/week for 3 weeks	*y_it_* = *μ_t_*+***γ**_zt_**z**_i_*+*α_i_*+*ε_it_*	*for*: *happiness*, *valued life*	*for*: *depression*, *anxiety*
			where *y_it_* is the outcome of interest (happiness, valued life, depression, or anxiety), and ***z**_i_* includes both *p* (prosocial acts condition) and *s* (self-focused acts condition). The effects of interest are:	*γ*_p1_ > 0	*γ*_p1_ < 0
				*γ*_p2_ > 0	*γ*_p2_ < 0
				*γ*_p3_ > 0	*γ*_p3_ < 0
		**Measurement intervals**: Respondents report activities on the three study days each week, and report happiness, valued life (an aspect of sense of meaning), depression, and anxiety at baseline, at the end of weeks 1, 2, 3, and at follow-up (week 5).			
				*γ*_p5_ > 0	*γ*_p5_ < 0
				Hypothesis 1b, 2b, 3b, and 4b confirmed if:	
Does prosocial behavior increase the sense of meaning in life?	2a. Prosocial acts will increase a sense of meaning relative to affectively neutral acts.				
			***γ***_*pt*_: the effects of *p* at each time point *t*		
	2b. Prosocial acts will increase a sense of meaning relative to self-focused acts intended to gratify personal emotional needs.				
		**Target Sample size**: 360 per condition x 3 conditions = 1080, based on a power analysis targeting 95% power (*α* = 0.05, 2-tailed tests)		*for*: *happiness*, *valued life*	*for*: *depression*, *anxiety*
			***γ***_*st*_: the effects of *s* at each time point *t*		
				*γ*_p1_ > *γ*_s1_	*γ*_p1_ < *γ*_s1_
			We will test all effects using Wald tests.	*γ*_p2_ > *γ*_s2_	*γ*_p2_ < *γ*_s2_
				*γ*_p3_ > *γ*_s3_	*γ*_p3_ < *γ*_s3_
				*γ*_p5_ > *γ*_s5_	*γ*_p5_ < *γ*_s5_
		**Sampling strategy**:			
		• sample in batches so sampling rate can be adjusted to hit target sample size–adjust based on observed attrition			
				We will count a hypothesis as confirmed if the associated parameter is statistically significant at *p* < 0.05. Note that hypotheses might be confirmed at some time points but not others.	
Does prosocial behavior reduce depression?	3a. Prosocial acts will reduce depression relative to affectively neutral acts.				
		• prevent responses from suspicious IP addresses			
		• replace responses that meet our data exclusion criteria			
	3b. Prosocial acts will reduce depression relative to self-focused acts intended to gratify personal emotional needs.				
Does prosocial behavior reduce anxiety?	4a. Prosocial acts will reduce anxiety relative to affectively neutral acts.				
	4b. Prosocial acts will reduce anxiety relative to self-focused acts intended to gratify personal emotional needs.				

### Ethics information

This study has been approved by the [name of ethics body redacted to allow double-blind peer review]. Informed consent will be obtained from all research participants prior to beginning the study. Participants will be paid for each component of the study that they complete. The compensation rate for each component is calculated based on its anticipated completion time, with a target pay rate of $14 CAD per hour (Ontario’s minimum wage). Study components include a baseline survey (15 min), nine daily surveys (3 min/each), and four follow-up surveys. The follow-up surveys are expected to take 10 minutes, but we will calculate pay rates based on 10 minutes (survey 1), 12.5 minutes (surveys 2 and 3), and 15 minutes (survey 4) to discourage attrition. In total, participants can earn $21 CAD ($16 USD) if they complete all components of the study.

### Design

We will examine the effects of prosocial behavior using a 3-week experimental intervention, followed by a follow-up assessment at 5 weeks. Tracking respondents over a 5-week period will provide insights into how durable prosocial effects are. This intervention will be embedded in a larger survey intended to address multiple questions regarding prosocial behavior and the COVID-19 pandemic. We focus solely on those parts of the survey relevant to the current, pre-registered intervention below.

**Fig 1:**
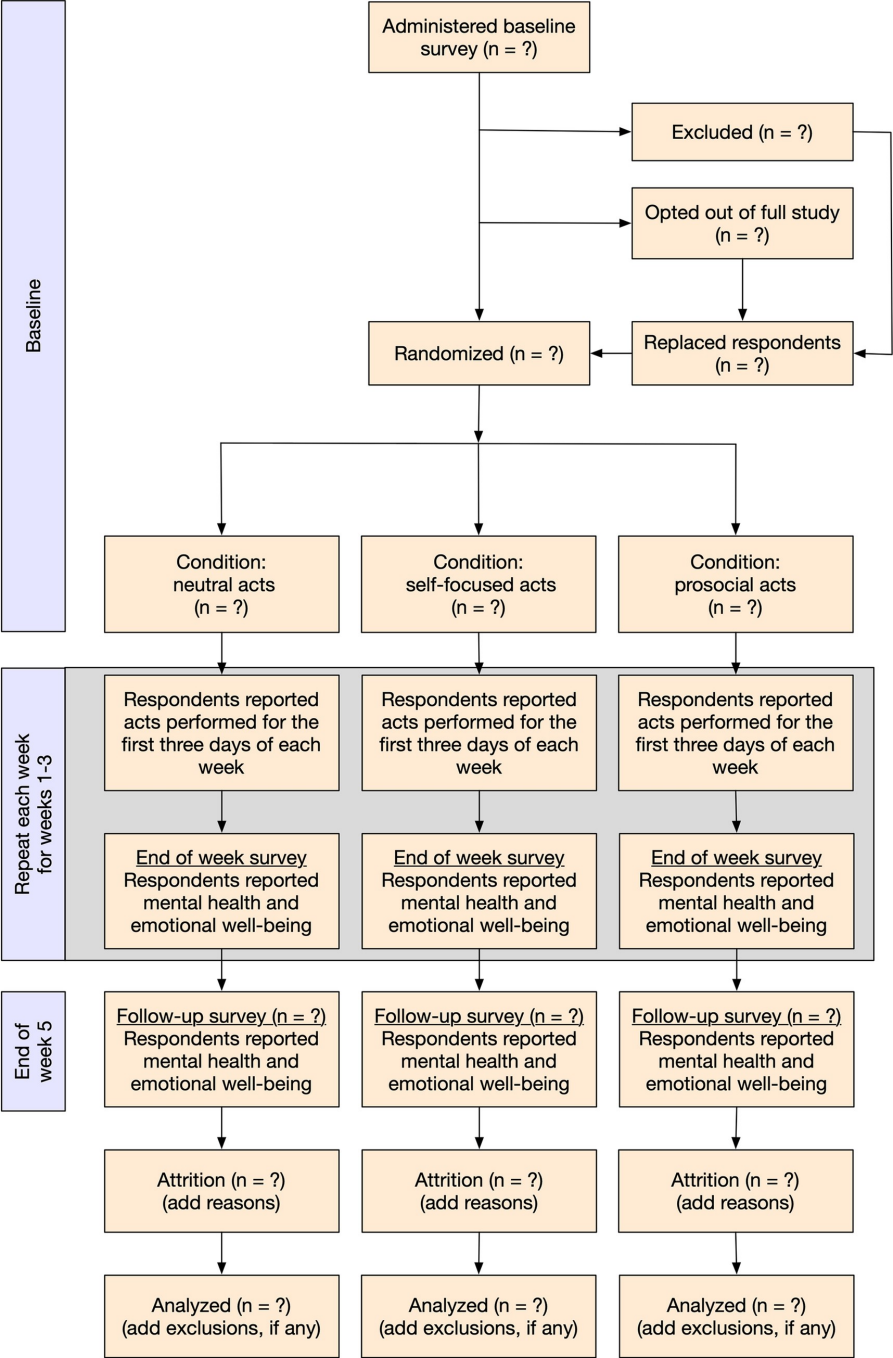
Flowchart of study procedures.

The study design is shown in Fig 1. At baseline we will measure participants’ emotional well-being and mental health. Emotional well-being will be assessed using happiness and feeling that one’s life is valuable, which is a facet of sense of meaning in life [58]. Mental health will be measured as depression and anxiety. At the end of the baseline survey, we will randomly assign participants to one of three experimental conditions (using the randomization feature in Qualtrics survey software; between subjects design; respondents will be blind to other conditions). In each condition, respondents will be asked to perform certain types of behaviors for the first three days of each week. There are several reasons we chose to assign behaviors on the first three days each week. First, it represents a compromise between existing study designs that ask respondents to perform acts every day, and those that ask them to perform multiple acts on a single day. Prosocial effects have been found using both study designs. We also believe that a three-times-a-week approach will be less burdensome for respondents and therefore increase study compliance. Keeping the acts on consecutive days (i.e., the *first* three days of each week rather than any three days) should make it easier for respondents to remember what to do and will simplify survey administration.

One possible concern is that the effect of our intervention will have worn off by the time we assess our outcomes at the week’s end, particularly since our models control for baseline levels of these variables. However, assessing effects at a delay is consistent with our aim to determine whether prosocial acts can make lasting contributions to mental health and emotional well-being. Further, specifying that respondents perform acts on the *first* three days of each week makes it clear *when* respondents are performing prosocial acts, making it easier to judge whether effects really do last over the course of several days.

For our experimental conditions, one group of participants will be asked to perform at least one prosocial act each day. This is our *prosocial intervention* condition. We will compare these respondents to two control conditions. In our *neutral control* condition, we will ask respondents to keep track of their daily activities, a task which we expect to be affectively neutral. Another group will be instructed to perform a personally enjoyable act each day. We expect this *self-focused* condition to produce positive emotions, but consistent with past research we suspect that it will be less effective at doing so than our prosocial intervention. A full list of experimental prompts for each of our three experimental conditions can be found in S2 Appendix.

Respondents will be drawn from Amazon’s Mechanical Turk and will be contacted using that platform’s built-in messaging capabilities for the first three days each week and asked to report what they did. At the end of weeks 1, 2, and 3 they will complete a longer survey that repeats the same measures of emotional well-being and mental health used at baseline. At this point the intervention will be complete. We will recontact respondent two weeks later (at the end of week 5) to assess whether the intervention has a lasting effect on mental health and emotional well-being. This final survey will also include a measure of whether or not respondents continued their assigned behaviors following the end of the intervention. This measure might be useful for explaining any lasting effects in exploratory analyses.

### Measures

#### Experimental condition

Experimental conditions will be coded using indicator variables for the prosocial intervention and self-focused conditions, with the neutral control as the reference category. We will use two commonly used indicators of *mental health*: depression and anxiety.

#### Depression

Depression will be measured using the well-established 8-item short-form of the Centre for Epidemiological Studies-Depression Scale (CES-D) [74]. We will ask respondents to report how often in the past week they (1) felt depressed, (2) felt that everything was an effort, (3) felt that sleep was restless, (4) felt happy (reverse coded), (5) enjoyed life (reverse coded), (6) felt lonely, (7) felt sad, and (8) could not get going. Responses will be scored where 0 = “rarely or none of the time,” 1 = “some of the time,” 2 = “a moderate amount of time,” and 3 = “most or all of the time.” We anticipate that this scale will have high alpha reliability scores [75, 76].

#### Anxiety

We will use the Hospital Anxiety and Depression Scale—Anxiety (HADS-A) scale to measure respondent anxiety, which is both commonly used and well-validated [77]. This is a 7-item scales that asks respondents how often in the past week they: (1) felt tense or wound up, (2) got a frightened feeling as if something awful was about to happen, (3) had worrying thoughts go through their mind, (4) got a frightened feeling like butterflies in the stomach, (5) felt restless as if they had to be on the move, (6) had a sudden feeling of panic, and (7) could sit at ease and feel relaxed. To ensure consistency with our measure of depression, responses will be coded where 0 = “rarely or none of the time,” 1 = “some of the time,” 2 = “a moderate amount of time,” and 3 = “most or all of the time.”

Both depression and anxiety will be coded so that higher scores indicate greater levels of mental distress.

We will also use two indicators of broader *emotional well-being*: subjective happiness and the sense that one’s life is valuable, which is a subset of the broader concept of a sense of meaning in life [58].

#### Subjective happiness

We will use the well-validated Subjective Happiness Scale [78]. The items are: (1) “In general, I consider myself _______.” Responses options run from 1 = “not a very happy person” to 7 = “a very happy person.” (2) “Compared to most of my peers, I consider myself ________” with response options from 1 = “less happy” to 7 = “more happy.” (3) “Some people are generally very happy. They enjoy life regardless of what is going on, getting the most out of everything. To what extent does this characterization describe you?” (1 = “not at all” to 7 = “a great deal”). (4) “Some people are generally not very happy. Although they are not depressed, they never seem as happy as they might be. To what extent does this characterization describe you?” (1 = “not at all” to 7 = “a great deal”; reverse coded) These four items will be averaged into a scale ranging from 1–7, where higher scores indicate greater subjective happiness.

#### Valued life

We will measure individual perceptions of whether one’s life has significance and value using the valued life subscale developed by Morgan and Farsides [74, [50]. This measure will consist of the average of the following four items: (1) “My life is worthwhile,” (2) “My life is significant,” (3) I really value my life,” and (4) I hold my own life in high regard.” In each instance, response options will run from -3 = “strongly disagree” to 3 = “strongly agree.” Steger et al. (2006) also offer a widely used meaning in life scale, but this scale only measures the coherence and purpose aspects of meaning in life and not the value a person places on his/her life. The Morgan and Farsides (2009) scale is also shorter than the Steger et al. scale (4 v. 10 items) making it more attractive from a survey administration standpoint.

All variables will be standardized prior to analysis, making it possible to directly compare the size of experimental effects across outcomes.

### Sampling plan

Respondents will be recruited from Canada and the United States using Amazon’s Mechanical Turk (MTurk). A short description of the study will be posted to the MTurk website. Potential respondents (registered MTurk workers) can click to read a longer study description (see S1 Appendix for recruitment materials). Those interested can then proceed to the study. The study will be open to all adults.

Research indicates that anywhere from 5% to 25% of responses collected using MTurk offer low quality data [79, 80]. Many of these responses come from respondents using virtual private servers (VPS) to access surveys they are not qualified for. Data quality can be substantially improved by blocking VPS users from taking surveys using techniques such as geolocation and IP address screening [79, 81]. Accordingly, we will collect data using the MTurk interface offered by CloudResearch which offers several data-quality safeguards. In particular, we will block participants that come from suspicious geocode locations (locations known or strongly suspected to be fraudulent) or that come from duplicate IP addresses. We will inform all potential respondents that we are blocking VPS users so that legitimate respondents who might otherwise use a VPS have the option of deactivating their VPS and accessing the survey. As added precautions, we will also use CloudResearch to filter out respondents whose IP addresses do not originate in the United States of Canada, and to block participants who have previously failed CloudResearch’s quality checks.

We are interested in the effects of prosocial acts on happiness, a sense that one’s life has value (valued life), depression, and anxiety. Few studies have examined the effects of prosocial behavior on valued life, depression, or anxiety. We therefore base our power calculations on studies of prosocial acts and happiness (including positive emotions). We draw estimates of effect sizes and sample sizes from a recent meta-analysis of prosocial behavior and emotions [20]. These estimates include the effects of prosocial acts compared to both neutral and self-focused behaviors. We excluded from consideration any effects from studies that sampled children or that used psychological flourishing as the outcome (as psychological flourishing is a broader concept than happiness). This left us with 37 effects.

Published studies of prosocial behavior likely suffer from small-sample bias [22]. To obtain a more accurate effect size for power calculations, we therefore used the “TOP10” heuristic proposed by Stanley et al. [82]. The TOP10 calculation is the average of the effect sizes in the top 10% of studies, where rankings are based on the reliability of the results. We used sample size as an indicator of study reliability. Applying TOP10 gives us 3.57 effects, which we round up to 4 to capture greater variety. These effects are: *d* = 0.08, 0.30, 0.20, and 0.18 [16, 68, 83]. The average of these four effects is *d* = 0.19. This is very similar to the estimate White et al. [22] give in their small-sample adjusted re-analysis of positive activity interventions (*r* = 0.10, which corresponds to *d* = 0.20).

We performed a power analysis to determine the necessary sample size to detect an effect size of *d* = 0.19 with 95% power. As described more fully below, our main analyses will include controls for baseline levels of all outcome variables. If we assume that these variables will account for at least 50% of the variance in our outcomes, then we need N = 357 or approximately 360 respondents per condition. The reasonableness of this assumption is indicated by large bivariate correlations between baseline and follow-up measures of emotional well-being and mental health in several studies. For example, Mongrain et al. [68] found that happiness measures correlated at 0.86 and depressive symptoms at 0.68 after one week. This indicates that the baseline measure of happiness alone would account for 0.86^2^ = 74% of the variance in happiness one week later, and that baseline depression would account for 0.68^2^ = 46% of the variance in depression one week later. Further, baseline happiness correlated with week 1 depression at -0.64, while baseline depression correlated with week 1 happiness at -0.63. This suggests that adjusting for baseline measures of *both* happiness and depression would almost certainly explain even more variation in week 1 measures. See studies such as Proyer et al. [84] and Manthey et al. [85] for similar correlations using different time frames. We expect a 30% attrition rate after baseline which means that we will sample 360/0.7 = 514 per condition [86].

We will post the baseline survey in two batches. The first batch will contain half the sample (N = 771), and we will use it to gauge the attrition rate (see data exclusion criteria below). We will then adjust the sample size of the second batch to try and capture the desired number of respondents per experimental condition. This will lower the size of the second batch if attrition rates are lower than expected or increase the size of the second batch if attrition rates are higher than expected. If we have not obtained a sufficient sample size at this point, we will repeat the process until we obtain at least 350 respondents in each experimental condition. To be clear, our stopping rule for recruiting sample participants does not require estimating any of the effects of interest in the study (i.e., it does not depend on the anticipated effect size). The only factor is whether we have obtained a sufficient number of respondents in each experimental condition.

Obtaining a sufficient per-condition sample size might take several days. We plan to begin sampling on a Sunday and continue sampling (if needed) on Monday. This means respondents will finish their first three days of the study on either Wednesday or Thursday. In either case, we will distribute the end of week 1 survey the following Sunday, which will then put both groups of respondents on the same schedule for the remainder of the study. We will try to recruit the full sample during this initial recruitment phase, as this will simplify administering the study. However, if our initial sampling does not yield a sufficient sample size, we will repeat the sampling procedure each week until we obtain a sufficient number of respondents. In this case, we will administer the study to different “cohorts” of respondents spaced a week apart.

#### Data exclusion criteria

Respondents will be excluded from the study if any of the following apply:

1. A respondent completes the baseline study unrealistically quickly. We measure response speed using the average number seconds spent on each survey item (seconds per item, or SPI). Following Wood et al. [80], we judge a response to be unrealistically fast if its SPI value is less than 1. SPI calculations will exclude optional items.
2. A respondent does not complete at least half of the items in the baseline survey.
3. A respondent provides off-topic, non-sensical (e.g., random or gibberish words), or non-English responses to an open-ended question in the baseline survey. The open-ended question follows a dictator game (that is not part of the pre-registered portion of the study) and asks respondents what they hoped to accomplish by acting as they did during the dictator game. The specific nature of this question will make it straightforward to detect off-topic responses. A comment must be judged as off-topic, non-sensical, or non-English by two members of the research team to be excluded.
4. A respondent does not agree to continue with the study when asked if they wish to continue taking part in the study at the end of the baseline study, or in private correspondence with the researchers.
5. A respondent completes the baseline study using an IP address from outside Canada or the United States, or that appears to originate from a VPS or other suspicious source. The CloudResearch platform should prevent these respondents from beginning the survey so we will not be actively checking location or VPS use. We include this exclusion criterion mainly as a precaution. If respondents are excluded based on this criterion, we will clearly document how we detected these respondents in the final report.
6. Technical difficulties prevent a respondent from completing the baseline study.

Because obtain sufficient power to detect effects is a central aim of this study, we will replace respondents who are removed for reasons 1–5 in a rolling fashion. In the case of technical failure (#6), our first approach will be to resolve the issue and administer the baseline survey to the same participant. If this is not possible, we will recruit a replacement.

#### Attrition during the study

We will count a respondent as having dropped out of the study if s/he fails to complete three surveys in a row (e.g., three daily surveys, two daily surveys and an end of week survey). We will assess attrition each week, and respondents who drop out will not be sent additional surveys. Respondents who drop out will be replaced in the next round of sampling unless they have completed at least one daily survey and one end-of-week survey. There are two reasons for this. First, this approach keeps the costs of the study predictable. Second, with some data for both daily and end of week surveys the full-information maximum likelihood missing data handling we employ will allow us to recover some of the power lost through sample attrition.

### Statistical analysis plan

Our goal is to determine the effects of acting prosocially on happiness, valued life, depression, and anxiety. Each of these outcomes will be measured at baseline, and at 1, 2, 3, and 5 weeks.

Past work has often assessed the effectiveness of prosocial interventions using t-tests or ANOVAs. However, these methods are inefficient when data exist that can explain a substantial amount of the variance in the outcome. Our data will contain baseline measures of all outcomes, which we expect to be highly related to our outcomes at all time points, and consequently capable of increasing the efficiency of our estimates. We will therefore include controls for baseline measures of all outcome variables in our models. If needed, we will also add controls for level of compliance with experimental instructions. At the end of week three, respondents will be asked to indicate how often they reported behaviors that they did not actually perform, and to indicate how much effort they put into performing acts that were beyond what they normally do (see S5 Appendix). One or both of these measures will be included as continuous covariates only if preliminary chi-square tests reveal that they are associated with experimental conditions.

For each outcome, we will estimate a random intercept model. Random intercept models adjust for the fact that each respondent contributes multiple data points to the analysis. These models will be of the form:

$$y_{it}=\mu_{t}+\gamma_{zt}z_{i}+\alpha_{i}+\varepsilon_{it}$$

Here *y*_*it*_ is the outcome variable that varies over both individuals (*i*) and time points (*t*). In this case, *t* can take on values of 1, 2, 3, and 5 indicating data from weeks 1, 2, 3, and 5. *μ*_*t*_ is an intercept that is allowed to vary over time, and **γ**_*zt*_ are the coefficients of the variables ***z***_*i*_ that are allowed to differ at each time point. The ***z***_*i*_ themselves are time-constant variables and so differ only between individuals. In this study ***z***_*i*_ incudes indicators for which experimental condition respondents were assigned to as well as controls for baseline measures of all mental health and emotional well-being variables. *α*_*i*_ allows the intercept for each individual to vary, and *ε*_*it*_ is a residual term that varies over both individuals and time.

**Fig 2:**
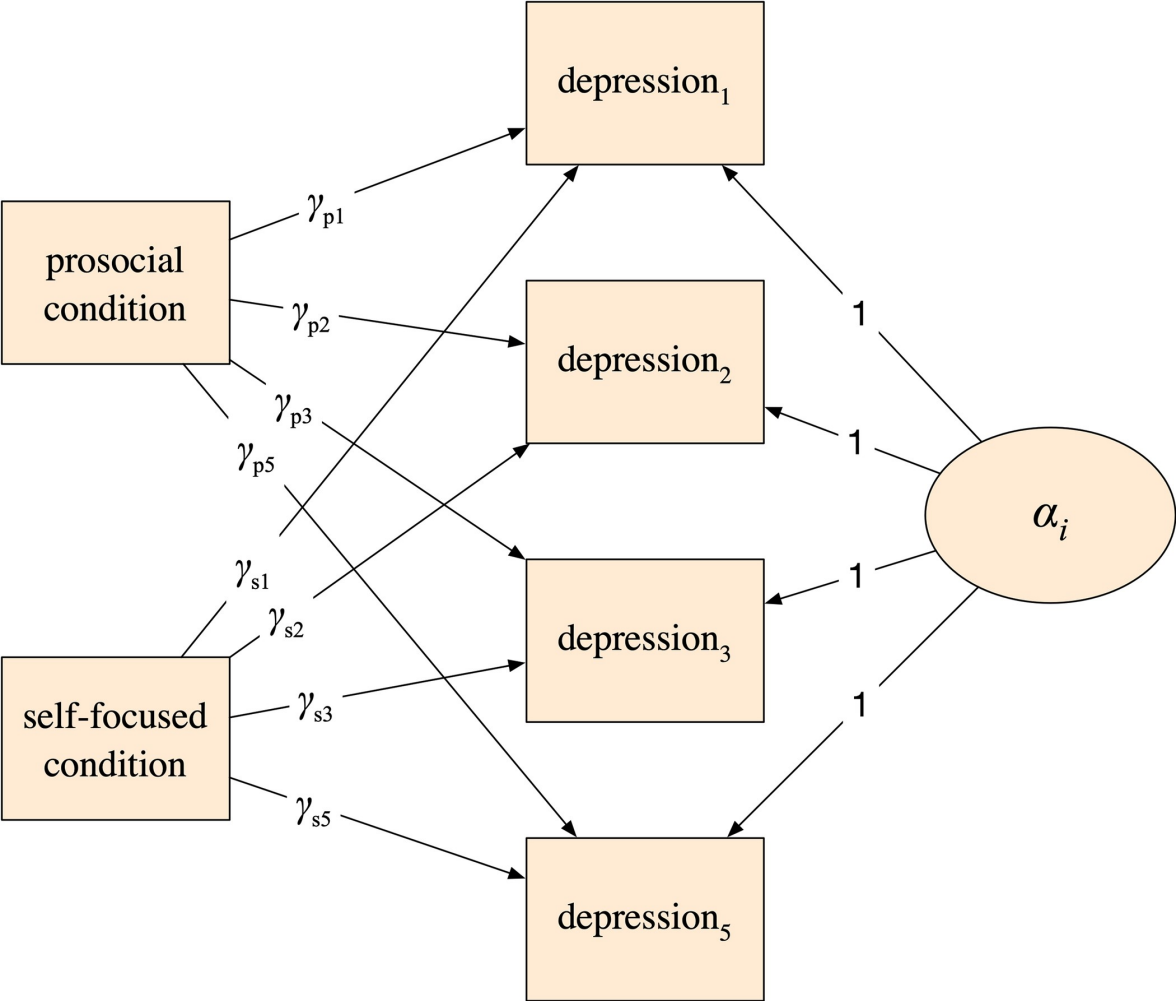
Example of a random intercept model as a structural equation model. Control variables, intercepts, and errors not shown for clarity.

We will estimate all random intercept models using structural equation modeling (SEM) software [87]. This will allow us to use full-information maximum likelihood (FIML) to adjust for missing data in all analyses [88]. As an example, Fig 2 displays the random intercept model for depression shown in SEM form (controls, intercepts, and errors not shown). The γ_*p*_*’*s are the effects of the prosocial intervention, and the γ_*s*_*’*s are the effect of the self-focused condition. This model is maximally flexible, estimating a unique effect of each condition at each time point. This is roughly equivalent to estimating a t-test at each time point, but with the added power afforded by our control variables. Analogous models can be drawn for the remaining outcome variables. Our study hypotheses can be evaluated by formally testing or comparing the coefficients from these models.

Table 2 lists the hypotheses we want to test for each outcome, and how those hypotheses map onto the model. To interpret the hypothesized direction of effects, recall that a beneficial effect means *greater* emotional well-being and *fewer* mental health challenges. We will test all hypotheses using Wald tests (i.e., the tests on individual coefficients, or post-estimation linear hypothesis tests).

**Table 2:** Hypotheses for testing intervention effects.

Hypothesis	Confirmed if	
	*for valued life*, *happiness*	*for depression*, *anxiety*
prosocial acts > neutral acts	*γ*_p1_ > 0	*γ*_p1_ < 0
	*γ*_p2_ > 0	*γ*_p2_ < 0
	*γ*_p3_ > 0	*γ*_p3_ < 0
	*γ*_p5_ > 0	*γ*_p5_ < 0
prosocial acts > self-focused acts	*γ*_p1_ > *γ*_s1_	*γ*_p1_ < *γ*_s1_
	*γ*_p2_ > *γ*_s2_	*γ*_p2_ < *γ*_s2_
	*γ*_p3_ > *γ*_s3_	*γ*_p3_ < *γ*_s3_
	*γ*_p5_ > *γ*_s5_	*γ*_p5_ < *γ*_s5_

We will count a hypothesis as confirmed if the associated parameter is statistically significant at *p* < 0.05. Note that hypotheses might be confirmed at some time points but not others (e.g., prosocial actions might have an effect on depression at week 1 but not at weeks 2, 3, or 5). While we acknowledge the possibility that effects might differ over time, we do not build predictions about changes over time into our hypotheses. This is consistent with the existing literature that has found prosocial effects following interventions of one to six weeks, and some work showing that these effects appear quickly and remain relatively constant over time [16].

All analyses will be performed in Stata version 16.

#### Planned exploratory analyses

A major motivation for these analyses is determining whether prosocial acts can safeguard mental health and emotional well-being during the COVID-19 pandemic. We accordingly will ask respondents to rate the impact the pandemic has had on their family life, social life (non-family), employment, leisure time, and financial situation. Response options will range from 1 = Very negatively affected to 7 = Very positively affected (midpoint = Not affected). We will then use moderation analyses (i.e., interaction terms) to test whether prosocial acts have the same effects for those affected by the pandemic as for those minimally affected by the pandemic.

We do not yet know how the COVID-19 impact data will be distributed, so it is difficult to know how best to code them and include them in analyses. Currently, we anticipate performing the following analyses. First, we will average the impact items to form a scale representing the overall impact of the COVID-19 pandemic. We will code respondents as affected by the pandemic if they score from 1 and 3 on the impact scale (very negatively affected to somewhat negatively affected), and as unaffected if they score between 3 and 5 (i.e., between somewhat negatively affected and somewhat positively affected, including those not affected). We do not anticipate many people being positively affected by the pandemic, so we do not include that category in our analyses. Second, to test moderation, we will re-estimate our analysis models and include interaction terms between an indicator for COVID-19 impact and each of our experimental condition variables. These terms will provide a formal test of whether prosocial effects differ between the two impact groups. We will calculate group-specific estimates using marginal effects. These analyses will provide insight into whether prosocial acts can be effective tools for improving emotional well-being both during and after the pandemic.

In the final report, we will update the text in this section as needed to reflect any changes to our exploratory analyses, and to accurately describe our decision processes. We anticipate introducing the exploratory analyses in general terms in the methods section, and then providing full details in the results section.

## Supporting information

S1 Checklist CONSORT 2010 checklist of information to include when reporting a randomised trial*. (DOC)

S1 File (ZIP)

S1 Appendix Recruitment materials for amazon’s mechanical turk. (DOCX)

S2 Appendix Prompts for prosocial intervention task. (DOCX)

S3 Appendix Questionnaire for baseline survey. (DOCX)

S4 Appendix Questionnaire for daily surveys. (DOCX)

S5 Appendix Questionnaire for end of week surveys. (DOCX)
