# Supplementary material for: Using prosocial behavior to safeguard mental health and foster emotional well-being during the COVID-19 pandemic: A registered report protocol for a randomized trial
Source: PLoS One. 2021 Jan 27;16(1):e0245865. doi: 10.1371/journal.pone.0245865 (PMC7840018; doi:10.1371/journal.pone.0245865)
Supplement: S1 File — (ZIP) [file pone.0245865.s002.zip › Guide to power analysis files.rtf]

These files run the power analysis in two ways:	1.	Using established formulas	2.	Using a simulation that mirrors our analysis modelThe calculations from each approach agree.
