# Supplementary material for: Using prosocial behavior to safeguard mental health and foster emotional well-being during the COVID-19 pandemic: A registered report protocol for a randomized trial
Source: PLoS One. 2021 Jan 27;16(1):e0245865. doi: 10.1371/journal.pone.0245865 (PMC7840018; doi:10.1371/journal.pone.0245865)
Supplement: S1 File — (ZIP) [file pone.0245865.s002.zip › power analysis using simulations.html]

Power simulations for prosocial behavior intervention pre-registration


# Power simulations for prosocial behavior intervention pre-registration

This document contains power calculations for the analyses planned for the COVID-19 prosocial behavior intervention study.

This specifies the random effects analysis model we will use.

```
modspec2 = "y1 ~ 1+gamma_p1*cond_p + gamma_s1*cond_s + cntrls0
y2 ~ gamma_p2*cond_p + gamma_s2*cond_s + cntrls0 
y4 ~ gamma_p4*cond_p + gamma_s4*cond_s + cntrls0
Alpha =~ 1*y1 + 1*y2 + 1*y4"
```

This function runs the simulation one time. It generates data, fits the model, and then returns the following:

- whether each parameter we are interested in is statistially significant
- the associated Cohen’s *d* value

Cohen’s *d* is a standardized value so it cannot be calcualted until after the model is fit. This is because the variance of the outcome depends on the simulation parameters.

The parameters are as follows:

- *g\_* parameters are all model coefficients. For instance, *g\_p1* is the effect of the prosocial condition on the outcome at week 1. *g\_s* coefficients are for the self-focused condition. *g\_c* effects are for the baseline controls.
- *alph* is the variance of the time-constant, individual-specific differences in the outcome
- *sd.e* effects are the idiosyncratic errors at each time point
- *just\_d* should the function just return the estimated Cohen’s *d*?
- *just\_p* should the function just return the estimated p-values?
- *return\_mod* should the function return the the fitted model object?

Note that this function simulates a two week intervention with a two week follow-up. This reflects an earlier verison of the study design. However, the method and results extend to a design of any number of weeks and so are still valid for the current study design.

```
run_sim1 = function(n, modspec=modspec2, g_p1=0, g_s1=0, g_c1=.99, alph=0.1, g_p2=NULL, g_s2=NULL, g_c2=NULL, g_p4=NULL, g_s4=NULL, g_c4=NULL, sd.e1 = 1, sd.e2=1, sd.e4=1, just_d = FALSE, just_p = FALSE, return_mod=FALSE) {
  #set parameters at weeks 2 and 4 to match those at week 1 unless otherwise specified
  if(is.null(g_p2)) g_p2 = g_p1
  if(is.null(g_p4)) g_p4 = g_p1
  if(is.null(g_s2)) g_s2 = g_s1
  if(is.null(g_s4)) g_s4 = g_s1
  if(is.null(g_c2)) g_c2 = g_c1
  if(is.null(g_c4)) g_c4 = g_c1
  
  #generate the data with specified simulation parameters

  #simulate data
  alpha = rnorm(n, sd=alph)
  whichcond = sample(c("prosocial", "self", "control"), size = n, replace=T)
  p = ifelse(whichcond=="prosocial", 1, 0)
  s = ifelse(whichcond=="self", 1, 0)
  c = ifelse(whichcond=="control", 1, 0)
  b = rnorm(n)
  e1 = rnorm(n, sd=sd.e1)
  e2 = rnorm(n, sd=sd.e2)
  e4 = rnorm(n, sd=sd.e4)
  y0=rnorm(n)
  y1 = g_p1*p + g_s1*s + g_c1*b + alpha + e1
  y2 = g_p2*p + g_s2*s + g_c2*b + alpha + e2
  y4 = g_p4*p + g_s4*s + g_c4*b + alpha + e4
  
  d = data.frame(cond_p = p,cond_s=s, cond_c = c,cntrls0=b,y0,y1,y2,y4)

  #fit the analysis model as an SEM  
  m = sem(modspec, data=d, se="robust")

  #extract and return all the coefficients/tests you are interested in, based on the things you want to test in the paper
  pe = parameterestimates(m)
  #extract results for other tests once we have them specified
  results = c(gamma_p1 = pe[pe$label=="gamma_p1","pvalue"],
              gamma_p2 = pe[pe$label=="gamma_p2","pvalue"],
              gamma_p4 = pe[pe$label=="gamma_p4","pvalue"],
              gamma_s1 = pe[pe$label=="gamma_s1","pvalue"],
              gamma_s2 = pe[pe$label=="gamma_s2","pvalue"],
              gamma_s4 = pe[pe$label=="gamma_s4","pvalue"]
              )
  sd_pool = sqrt((sd(d$y1[d$cond_p==1])^2 + sd(d$y1[d$cond_c==1])^2)/2)
  cohen_d = (mean(d$y1[d$cond_p==1]) - mean(d$y1[d$cond_c==1]))/sd_pool
  if(return_mod) return(list(cohen_d = cohen_d, param_p = results, mod = m))
  if(just_d) return(cohen_d)
  if(just_p) return(results)
  return(list(cohen_d = cohen_d, param_p = results))
  
} #end run_sim1()
```

The first task is to get a model that approximates our assumption that the baseline effects will explain roughly half the variation in the outcome. This require testing different plausible model parameters until one returns an \(r^2\) value of 0.50. For simplicity, we just present the final value we used.

```
varvals = mclapply(rep(10000, 1000), run_sim1, return_mod=T, mc.cores=8)
varvals = lapply(varvals, 
                 function(x) return(lavInspect(x$mod, "rsquare")["y1"])
                 )
summary(unlist(varvals))
```

```
##    Min. 1st Qu.  Median    Mean 3rd Qu.    Max. 
##  0.4716  0.4924  0.4978  0.4978  0.5031  0.5249
```

The defaults in the `run_sim1()` function do the job.

Given this baseline assumption, what raw coefficient value for the prosocial experimental condition gives us a Cohen’s *d* of 0.19?

We’ll run the simulation on a large data set to see what the median value is. Again, we just present the final simulation value that we used.

```
dvals = mclapply(rep(10000, 1000), run_sim1, just_d=T, g_p1=.268, mc.cores=8)
summary(unlist(dvals))
```

```
##    Min. 1st Qu.  Median    Mean 3rd Qu.    Max. 
##  0.1177  0.1720  0.1894  0.1894  0.2053  0.2732
```

Given these assumptions and simulation values, determine the sample size needed for 95% power.

```
get_pwr = function(sampsize, nreps, alpha=0.05, numcores=4, ...) {
    pvals = mclapply(rep(sampsize, nreps), run_sim1, just_p=TRUE, ..., mc.cores=numcores)
    pvals = lapply(pvals, function(x) return(x[1]))
    pvals = unlist(pvals)
    return(mean(pvals < alpha, na.rm=TRUE))
} #end get_pwr()
```

The sample size that gets us about 95% power is 1080, or roughly 360 per experimental condition.

```
get_pwr(sampsize=1080, nreps=2000, g_p1=.268, numcores=8)
```

```
## [1] 0.949
```

This sample size is very close to the value obtained using the formulas.
