# Supplementary material for: Using prosocial behavior to safeguard mental health and foster emotional well-being during the COVID-19 pandemic: A registered report protocol for a randomized trial
Source: PLoS One. 2021 Jan 27;16(1):e0245865. doi: 10.1371/journal.pone.0245865 (PMC7840018; doi:10.1371/journal.pone.0245865)
Supplement: S1 File — (ZIP) [file pone.0245865.s002.zip › power analysis using formulas.html]

Power Calculation for Prosocial Behavior Intervention


# Power Calculation for Prosocial Behavior Intervention

This file details how we calculated statistial power for our prosocial behavior intervention study.

First, we employed the TOP10 heuristic to obtain the anticipated effect size, as detailed in the Stage 1 protocol. This gave an effect size of *d* = 0.19. We then converted this effect to the \(r^2\) metric using the calculator found here: https://www.polyu.edu.hk/mm/effectsizefaqs/calculator/calculator.html. This gives a value of \(r^2 = 0.00894428\).

We then converted the \(r^2\) value to an \(f^2\) value using the formula:

\(f^2 = \frac{r\_{AB}^2 - r\_A^2}{1 - r\_{AB}^2}\)

where \(r\_A^2\) is the variance explained by the model without the experimental effect and \(r\_{AB}^2\) is the variance explained by the model with the experimental effect included (see https://www.statmethods.net/stats/power.html). We assume that our baseline measures of all outcomes will explain at least 50% of the variance in the outcome as explained in the baseline protocol. This means that \(r\_A^2 = 0.50\). The experimental effect explains an additional 0.00894428, so \(r\_{AB}^2 = 0.50894428\).

```
f2 = (0.50894428 - 0.50)/(1-0.50894428)
```

This gives us an \(f^2\) value of 0.0182144.

We next used our \(f^2\) value to calculate the necessary sample size to detect a single experimental effect with 95% power. This requires the *pwr* package.

```
pwr::pwr.f2.test(f2=f2, u=1, power=.95)
```

```
## 
##      Multiple regression power calculation 
## 
##               u = 1
##               v = 713.3575
##              f2 = 0.01821439
##       sig.level = 0.05
##           power = 0.95
```

The quantity *v* gives the sample size necessary to detect a single experimental effect—that is, one experimental condition compared to one control condition. Thus the per condition sample size is 714/2 = 357.
