# Supplementary material for: Using prosocial behavior to safeguard mental health and foster emotional well-being during the COVID-19 pandemic: A registered report protocol for a randomized trial
Source: PLoS One. 2021 Jan 27;16(1):e0245865. doi: 10.1371/journal.pone.0245865 (PMC7840018; doi:10.1371/journal.pone.0245865)
Supplement: S1 Appendix — (DOCX) [file pone.0245865.s003.docx]

**S1 Appendix. Recruitment Materials for Amazon’s Mechanical Turk**

**MTURK STUDY SHORT DESCRIPTION**

This survey is the first part of a 3-week study about behaviors, emotions, and mental health. Participants who complete the full 3-week study will earn $16.

**MTURK STUDY LONG DESCRIPTION**

This survey is the first part of a 3-week study of behaviors, emotions, and mental health. We’ll first collect some basic information about you, and then ask you to try out some simple behaviors a few times a week for 3 weeks. One each of those days, we’ll send you a short survey so that you can tell us what you did, and how you feel. We’ll send you longer “check-in” surveys at the end of weeks 1, 2, and 3. We’ll also send you a follow-up survey two weeks after the study ends. All surveys will be administered through Mechanical Turk, and you’ll be paid for each survey you complete. In total, you can earn up to $16. We’ll send you an email reminder each day.

If you wish to take part in this study, please read the following consent form and then click below to proceed to the first survey.

Warning! This survey uses a protocol to check that you are responding from inside the United States or Canada and not using a Virtual Private Server (VPS), Virtual Private Network (VPN), or proxy to hide your country. In order to take this survey, please turn off your VPS/VPN/proxy if you are using one and also any ad blocking applications. Failure to do this might prevent you from completing the HIT. For more information about why we are requesting this, see this post from CloudResearch (https://goo.gl/WD6QD4).

**Keywords**: mental health, behavior, emotions, survey, task, happiness.

*NOTE: The VPS warning was adapted from Kennedy et al.* [1].

**References**

1. Kennedy R, Clifford S, Burleigh T, Waggoner PD, Jewell R, Winter NJG. The shape of and solutions to the MTurk quality crisis. Polit Sci Res Methods. 2020; 1–16. doi:10.1017/psrm.2020.6
