# Supplementary material for: Using prosocial behavior to safeguard mental health and foster emotional well-being during the COVID-19 pandemic: A registered report protocol for a randomized trial
Source: PLoS One. 2021 Jan 27;16(1):e0245865. doi: 10.1371/journal.pone.0245865 (PMC7840018; doi:10.1371/journal.pone.0245865)
Supplement: S2 Appendix — (DOCX) [file pone.0245865.s004.docx]

**S2 Appendix. Prompts for Prosocial Intervention Task**

You are almost done with this survey. Now comes the most important part, so please read carefully!

We are going to ask you to do something that could increase your happiness.

[INSERT PROMPT HERE, RANDOMLY ASSIGNED]

| Condition | Prompt |
| --- | --- |
| 1. Neutral control | For the next three weeks, we would like you to keep track of the things you do. Do not alter your routine in any way: simply keep track of what you do. Do this for the first three days of each week, starting tomorrow. You are welcome to continue tracking your activities after those three days, but please be sure to do track your activities on at least those three days.    We’ll send you a quick 2-3 minute survey each day so that you can tell us what you did, and a longer “check-in” survey at the end of each week. For the purposes of this study, the first week begins tomorrow regardless of what day it is.    To start you thinking, please list several activities that you might do on a typical day that you do not find taxing or unpleasant.    [SPACE TO ENTER UP TO FIVE ACTS]    Again, please keep track of your activities each day for the next three days. We understand that life is busy so you might occasionally miss a day, but please try your best. |
| 2. Self-focused | For the next three weeks, we would like you to “treat yourself” by doing things that you enjoy. These acts don’t have to be large or costly, but they should be over and above what you typically do. Do one (or more) things you enjoy each day for the first three days of each week, starting tomorrow. You are welcome to continue doing enjoyable things after those three days, but please be sure to do enjoyable things on at least those three days.    We’ll send you a quick 2-3 minute survey each day so that you can tell us what you did, and a longer “check-in” survey at the end of each week. For the purposes of this study, the first week begins tomorrow regardless of what day it is.    To start you thinking, please list several things that you might want to do to treat yourself in the coming three weeks.    [SPACE TO ENTER UP TO FIVE ACTS]    Again, please do one (or more) things that you enjoy each day for the next three days. We understand that life is busy so you might occasionally miss a day, but please try your best. |
| 3. Prosocial intervention | For the next three weeks, we would like you to perform acts of kindness.    By acts of kindness, we mean behaviors that benefit someone else and are over and above what you typically do (i.e., they are not expected of you). These acts should also involve some sacrifice by you (e.g., in effort, energy, time, or money). Do one (or more) kind acts each day for the first three days of each week, starting tomorrow. You are welcome to continue doing kind acts after those three days, but please be sure to do kind things on at least those three days.    We’ll send you a quick 2-3 minute survey each day so that you can tell us what you did, and a longer “check-in” survey at the end of each week. For the purposes of this study, the first week begins tomorrow regardless of what day it is.    To start you thinking, please list several different kind acts that you might want to do in the coming three weeks.    [SPACE TO ENTER UP TO FIVE ACTS]    Again, please do one (or more) kind acts each day for the next three days. We understand that life is busy so you might occasionally miss a day, but please try your best. |

*NOTE:* *The following experimental prompts were adapted from Sheldon et al.* [1] *and Nelson et al.* [2]

**References**

1. Sheldon KM, Boehm J, Lyubomirsky S. Variety is the Spice of Happiness: The Hedonic Adaptation Prevention Model. In: Boniwell I, David SA, Ayers AC, editors. Oxford Handbook of Happiness. 2013.

2. Nelson SK, Layous K, Cole SW, Lyubomirsky S. Do unto others or treat yourself? The effects of prosocial and self-focused behavior on psychological flourishing. Emotion. 2016;16: 850–861. doi:10.1037/emo0000178
