# Supplementary material for: Using prosocial behavior to safeguard mental health and foster emotional well-being during the COVID-19 pandemic: A registered report protocol for a randomized trial
Source: PLoS One. 2021 Jan 27;16(1):e0245865. doi: 10.1371/journal.pone.0245865 (PMC7840018; doi:10.1371/journal.pone.0245865)
Supplement: S4 Appendix — (DOCX) [file pone.0245865.s006.docx]

**S4 Appendix. Questionnaire for Daily Surveys**

ACT1. Please describe [one thing that you did today/one thing you did to treat yourself/a kind act you performed] in the space below. Describe what you did. [for those in the experimental conditions] If you forgot to [do something to treat yourself/do a kind act], just tell us below and try again tomorrow.

[Text box]

ACT2. List anything else you did that you’d like to report. If you didn’t do anything else, skip to the next question.

[Text box]

*NOTE: This questionnaire is an edited version of the full questionnaire that focuses on just those parts of the project that are relevant to the preregistered study. Other measures designed for subsequent exploratory analyses are not included here.*
