# Supplementary material for: Using prosocial behavior to safeguard mental health and foster emotional well-being during the COVID-19 pandemic: A registered report protocol for a randomized trial
Source: PLoS One. 2021 Jan 27;16(1):e0245865. doi: 10.1371/journal.pone.0245865 (PMC7840018; doi:10.1371/journal.pone.0245865)
Supplement: S5 Appendix — (DOCX) [file pone.0245865.s007.docx]

**S5 Appendix. Questionnaire for End of Week Surveys**

*End of Week 1, End of Week 2, End of Week 3 survey*

Include these measures from the baseline survey:

Subjective Happiness Scale

Sense of Purpose and Meaning

Depression and Anxiety

[IN WEEK 1 AND WEEK 2 SURVEYS ONLY]

Just a reminder that tomorrow starts the next week of the study. That means you should [CONDITION SPECIFIC PROMPT – adapted from original experimental prompts] each day for the next three days. We’ll send you a short 2-3 minute survey each of those days.

That’s it! Enter your survey code to receive [ENTER PAYMENT AMOUNT] for completing this survey. Just FYI, the survey at the end of next week will pay [ENTER PAYMENT AMOUNT].

[AT END OF WEEK 3 SURVEY ONLY]

**The next three questions will not affect your payment. We only ask so that we can better understand your responses and interpret our data appropriately.**

ACTS_REPORT. For the last three weeks we’ve asked you to [CONDITION SPECIFIC PROMPT], and to report what you did to us. We realize that this could be challenging to do consistently, so we’d like to see now how you did. Please read the following options and select the one that best represents what you did.

1. I never actually performed any of the behaviors I reported
2. I frequently reported behaviors that I did not actually perform
3. I occasionally reported behaviors that I did not actually perform
4. I only reported behaviors that I had actually performed
5. Other (please specify) [TEXT BOX]

| **Condition** | **Prompt** |
| --- | --- |
| 1: Control | keep track of the things you did |
| 2: Enjoyable acts | “treat yourself” by doing things that you enjoy |
| 3: Prosocial acts | do kind acts |

[SHOW IF ACTS_REPORT > 1]

ACTS_EFFORT. You were asked to [CONDITION SPECIFIC PROMPT]. Which of the following best describes how you tried to complete this task?

1. I did nothing beyond what I normally do—I just reported behaviors I happened to have performed that day
2. I occasionally tried to do something beyond what I normally do
3. I often tried to do something beyond what I normally do
4. I always tried to do something beyond what I normally do
5. Other (please specify) [TEXT BOX]

ACTS_VARIETY. Think again about the acts you reported. How often did you vary what you did from day to day?

1. Never – I did the same thing every day
2. A few times
3. About half the time
4. Most of the time
5. All the time – I did something different every day

That’s it! You have finished the study! We will send you a follow-up survey in about two weeks to see how you are doing.

*End of Week 5 survey*

This survey is a follow-up to a 3-week study you finished 2 weeks ago. It should take you about 15 minutes to complete.

Include these measures from the baseline survey:

Subjective Happiness Scale

Sense of Purpose and Meaning

Depression and Anxiety

CONT_ACTS. During the study we asked you to [INSERT CONDITION SPECIFIC PROMPT]. How often have you continued to [INSERT CONDITION SPECIFIC PROMPT] during the two weeks since the study ended?

0 = Never

1 = Once or twice

2 = On about half the days

3 = Nearly every day

4 = Every day

| **Condition** | **Prompt** |
| --- | --- |
| 1: Control | keep track of the things you did |
| 2: Enjoyable acts | “treat yourself” by doing things that you enjoy |
| 3: Prosocial acts | do kind acts |

*NOTE: This questionnaire is an edited version of the full questionnaire that focuses on just those parts of the project that are relevant to the preregistered study. Other measures designed for subsequent exploratory analyses are not included here.*
